# Community views on short birth interval in Northern Uganda: a participatory grounded theory

**DOI:** 10.1186/s12978-021-01144-5

**Published:** 2021-04-28

**Authors:** Loubna Belaid, Pamela Atim, Emmanuel Ochola, Bruno Omara, Eunice Atim, Martin Ogwang, Pontius Bayo, Janet Oola, Isaac Wonyima Okello, Ivan Sarmiento, Laura Rojas-Rozo, Kate Zinszer, Christina Zarowsky, Neil Andersson

**Affiliations:** 1 CIET (Community Information Epidemiological Technologies), Department of Family Medicine (PRAM), McGill University, 5858 Chemin de la Côte des Neiges, Montreal, QC H3S 1Z1 Canada; 2 Department of Public Health, Gulu University, Laroo Division, Gulu Municipality 166, Gulu, Uganda; 3 St Mary’s Lacor Hospital, Gulu/P.O. Box, 180, Gulu, Uganda; 4 Gulu University, Gulu Municipality 166, Gulu, Uganda; 5 Nwoya Health District, Gulu, Uganda; 6 Department of Family Medicine, McGill University, 5858 Chemin de la Côte des Neiges, Montreal, QC H3S 1Z1 Canada; 7 University of Montreal, 7101 Av du Parc, Montreal, QC H3N 1X9 Canada; 8 Centro de Investigación de Enfermedades Tropicales (CIET), Universidad Autónoma de Guerrero, Acapulco, Mexico

**Keywords:** Short birth intervals, Gender dynamics, Northern Uganda, Participatory research, Grounded emic theory, Community-led solutions

## Abstract

**Background:**

Short birth interval is associated with adverse perinatal, maternal, and infant outcomes, although evidence on actionable factors underlying short birth interval remains limited. We explored women and community views on short birth intervals to inform potential solutions to promote a culturally safe child spacing in Northern Uganda.

**Methods:**

Gendered fuzzy cognitive mapping sessions (n = 21), focus group discussions (n = 12), and an administered survey questionnaire (n = 255) generated evidence on short birth intervals. Deliberative dialogues with women, their communities, and service providers suggested locally relevant actions promote culturally safe child spacing.

**Results:**

Women, men, and youth have clear understandings of the benefits of adequate child spacing. This knowledge is difficult to translate into practice as women are disempowered to exercise child spacing. Women who use contraceptives without their husbands’ consent risk losing financial and social assets and are likely to be subject to intra-partner violence. Women were not comfortable with available contraceptive methods and reported experiencing well-recognized side effects. They reported anxiety about the impact of contraception on the health of their future children. This fear was fed by rumors in their communities about the effects of contraceptives on congenital diseases. The women and their communities suggested a home-based sensitization program focused on improving marital relationships (spousal communication, mutual understanding, male support, intra-partner violence) and knowledge and side-effects management of contraceptives.

**Conclusions:**

The economic context, gender power dynamics, inequality, gender bias in land tenure and ownership regulations, and the limited contraceptive supply reduce women’s capacity to practice child spacing.

**Supplementary Information:**

The online version contains supplementary material available at 10.1186/s12978-021-01144-5.

## Plain language summary

The evidence on what increases birth spacing remains limited. This study explored community views on influences on short birth interval to promote a culturally safe child spacing in Northern Uganda. A participatory research process began by collating perspectives of causes of short birth intervals through fuzzy cognitive mapping. Focus group discussions clarified concepts emerging from the fuzzy cognitive mapping exercise. Fieldworkers administered a household survey to quantify reproductive health outcomes. In deliberative dialogue sessions involving women and their communities, shared and discussed these results and suggested potential actions to promote culturally safe child spacing. Women, men, and youth showed clear understandings of the benefits of adequate child spacing. This knowledge is difficult to translate into practice, however, as women feel they are unable to exercise child spacing. Women who use contraceptives without their husbands’ consent risk losing financial and social resources and are likely to face intra-partner violence. Women were not comfortable with contraceptive methods and reported experiencing side effects. The deliberative dialogues suggested a home-based sensitization program focused on improving marital relationships (spousal communication, mutual understanding, male support, intra-partner violence) and knowledge and side-effects management of contraceptives.

**Supplementary Information:**

The online version contains supplementary material available at 10.1186/s12978-021-01144-5.

## Introduction

The World Health Organization recommends a birth interval of at least 24 months from one live birth to the subsequent pregnancy to reduce the risk of adverse maternal, perinatal, and infant outcomes [1]. In practice, this corresponds to 33 months birth interval. Short birth intervals are associated with pre-term delivery, low-birth weights or small for gestational age neonates, and obstetric complications like premature rupture of membrane, *abruptio placentae,* and *placenta previa* [2, 3].

Globally, 24.6% of births occur at intervals of less than 24 months. Sub-Saharan Africa has a short birth interval of 19.6% compared with 24.7% in South Asia [4]. Short birth intervals were not measured in the latest Ugandan Demographic Health Survey (DHS), but the median birth interval increased from 29.2 months in 2000–01 to 31.9 months, and about one in four non-first births (24%) occurred less than 24 months after the preceding birth [5]. In Northern Uganda, the Acholi sub-region has a median birth interval of 32.9 months [5].

While there is a large body of evidence on factors associated with family planning uptake services in sub-Saharan Africa [6–8], limited literature examined the views on short birth interval [9–11]. A 2020 mixed-methods systematic review on short birth interval found that the mother’s education and age, previous pregnancy outcome, breastfeeding, contraception, socioeconomic level, parity, and gender of the preceding child are commonly explored. These studies reported mixed results (positive or negative) for most factors associated with short birth interval. A shorter breastfeeding period and the female gender of the previous child were the only factors consistently associated with short birth intervals [9].

A 2015 study, based on a secondary analysis of the 2011 Ugandan DHS, reported women in communities with lower levels of unmet need were significantly less likely to report birth intervals of less than 24 months than 25–38 months [11]. Women who resided in wealthier communities were more likely to report longer birth intervals of 25–38 months [11]. A 2020 cross-sectional study in rural Uganda found that young age of motherhood, not using contraceptives, and lack of male involvement were associated with short birth intervals [10]. While these studies suggest several potential causes of short birth intervals, they are not necessarily actionable everywhere. Grounding in the local setting can increase the relevance of any intervention. This study explored women and community views about the causes of short birth intervals to inform a culturally safe approach to child spacing in Nwoya district in northern Uganda.

## Methods

### Study setting

The project involved three parishes in Nwoya district in the Acholi subregion of Northern Uganda, including some 214,000 people [12]. Most of the population is rural and depends on subsistence-based agriculture. Northern Uganda went through two decades of a civil war between 1986 and 2006. Armed conflict with the Lord’s Resistance Army displaced about 90% of the population to camps of internally displaced persons with disruption of public resources, loss of land, and deterioration of reproductive health indicators [13].

The northern region now has one of the highest maternal mortality levels, neonatal mortality, and teenage pregnancy in the country [5]. The national maternal mortality ratio (MMR) is 336 maternal deaths per 100,000 live births, and almost 28% of maternal deaths occur among women between 15 and 24 years of age. The MMR Northern Uganda, the MMR is near twice this (650 women in every 100,000 live births), and at least ten times this number of children dies before 1 month of age [14]. The maternity risks start early in life, with one in every four women beginning childbearing by the age of 19 years [5].

### Study design

This study is part of a larger cross-sectional, mixed-methods participatory research initiative that engaged women and their families to work with service providers to improve perinatal care access and promote culturally safe child spacing practices [15]. The participatory research perspective sees women and their communities as knowledge holders and partners in co-design. The grounded theory focuses on the emic perspective and provides inductive strategies for building theories from within the participant’s worldview [16, 17]. The substantive theory that emerged from their collective knowledge informed interventions to promote a culturally safe child spacing approach, implementation of which is outside the scope of this report.

### Data collection

#### Overview

Fuzzy cognitive mapping (FCM) collated views of women and their communities' views on the causes of short birth intervals. Subsequent focus group discussions clarified some of the concepts that emerged from the FCM. An administered questionnaire in a household survey quantified variables on reproductive and family planning outcomes. Deliberative dialogue sessions with women and their communities shared and discussed the results. They suggested potential actions to promote culturally safe child spacing. We used data from these processes to build the emic grounded theory.

#### Fuzzy cognitive mapping

FCM aims to understand stakeholder knowledge and views on causes, in this case, of short birth intervals. Participants identified links between causal factors and outcomes and assigned a weight to each one based on their perceptions of its relative importance [18, 19]. We purposively sampled seven stakeholder groups: women, men, youth (18–25 years old) (women and men), community health workers, traditional midwives, and service providers. We invited five to eight participants from each group for each session. We met each stakeholder group separately, convening one group per stakeholder category and parish. In each session, participants drew two maps. One map identified child spacing benefits and the other map described the causes of the short birth intervals. Local female trained researchers convened participants for the mapping sessions. One researcher facilitated the sessions in the Acholi language, and the other one took systematic notes.

#### Focus groups discussion

We conducted focus groups to explore the concepts that emerged from the mapping sessions. A female anthropologist part of the research team designed the focus group guide. We invited participants by phone. The local female researchers moderated the groups with the same women, men, and youth who participated in the mapping sessions. We conducted 12 sessions (three women, three women youth (18–25 years old), three men, three men youth), each group with 5–8 participants. The local researchers met groups separately. Each focus group lasted 1 h in the Acholi language and was held in public places (outside near a health center or a school). We did not record the proceedings to allow the participants to speak freely [20, 21].

#### Survey

The questionnaire collated socio-demographic characteristics and gynecology history: age, number of children, women education and occupation, husband occupation, agriculture land and size, decision on land issues, children external support, household sources of income, food, known risk factors: knowledge on danger signs for pregnancy, and childbirth, blurred vision, dizziness, repeated headaches, verbal, mental, physical abuse, outcomes for the most recent pregnancy: male support, transport to health facility, antenatal check-ups, blood and urine tests, childbirth in facility, post-partum: fever within the 6 weeks after delivery, foul discharge within 6 weeks after delivery, and contraception: attitude towards modern contraceptives, contraceptive currently used, capability to use modern contraceptives, factors affecting decision on spacing children.

The local team pretested the e-questionnaire among women who had given birth in the past 2 years. We used a cluster sampling method to select the villages in each parish. We used Android tablets and Open Data Kit (ODK) software to administer the questionnaires. ODK is an open-source software to collect data offline at scale. The duration of administering the questionnaire was approximately 30 min.

#### Deliberative dialogue

Deliberative dialogue is a reflective evidence-based discussion where members from each stakeholder group listen to each other’s interpretation of the evidence before identifying, in this case, ways to promote culturally safe child spacing. The participants discussed the findings from the mapping and focus groups [22]. Local researchers facilitated three deliberative dialogue sessions in Acholi, one in each parish and each attended by 15–20 participants. Each deliberative dialogue lasted 3–4 h (Table 1).

**Table 1:** Synthesis of data collection methods

Data collection	Gender and age	Number	Selection criteria
Fuzzy cognitive mapping (FCM)	Men and women ≥ 18 year old	5–8 participants for 7 groups, per parish Total: 21 FCM sessions	Separate sessions with homogenous groups of men (youth/ adult), women (youth/ adult) TMs, CHWs service providers in each parish
Focus groups discussion (FGD) participants	Men and women ≥ 18 year old	5–8 participants Total: 12 FGD	Separate sessions with homogenous groups of men (youth/ adult), women (youth/ adult) in each parish
Deliberative Dialogues (DD) participants	Men and women ≥ 18 year old	DD each 15–20 participants Total: 3 sessions	Heterogeneous sessions with participants who joined the FCM and FGDs sessions from each stakeholder groups
Survey participants	Women ≥ 18 year old	Women who have delivered in the last 2 years in the 3 parishes	

### Data management and analysis

We digitized the fuzzy cognitive maps using yEd software [23]. Based on a thematic approach, we condensed the factors from the maps into fewer categories using Excel sheet tables (Additional file 1: Appendix S1a, Additional file 2: Appendix S1b). Transitive closure analysis identified the most influential factors in the maps and a pattern-matching table showed similarities and differences across stakeholder groups (Additional file 3: Appendix S2a, Additional file 4: Appendix S2b). We aggregated the weights from combined maps and calculated the cumulative influence as the sum of all the weights. The final map scaled the cumulative influences dividing them by the maximum cumulative influence in the map to obtain values between 0 and 1. We used Ciet map 2.2, a windows-like interface for R, to conduct the transitive closure analysis.

The local researchers translated the focus groups and deliberative dialogue notes into English. Using an inductive thematic analysis and an open coded approach, we constructed the code structure based on a sample of transcriptions [24]. We consolidated the coding structure and coded the transcriptions manually. We looked for patterns, similarities, and contrasts between women, men, youth, and parishes.

The survey provided baseline indicators, response rates, and variances for calculating sample size for a future impact evaluation study. We tested the handset tablet application in this setting, including training, quality control, data collation, and cleaning. This survey was not powered to measure impact but settled the feasibility and acceptability issues. We used Excel to quantify reproductive outcomes and complement the mapping and focus groups' data. The mapping and focus groups' findings built the emic theory, and the data from the deliberative dialogue enriched the grounded theory on short birth intervals. The female anthropologist drafted this narrative report.

#### Ethics

This study respected the principles in the Declaration of Helsinki. We obtained ethical approvals from the Uganda National Council for Science and Technology (SS479) and the Faculty of Medicine and Health Sciences of McGill University (A08-B01-19A) in Canada. We sought informed verbal consent from the participants. We treated all information from participants as confidential.

## Results

Fuzzy cognitive mapping sessions clarified the meaning of child spacing with two maps (child spacing benefits and causes of short birth intervals). Participants viewed child spacing as:

“*Child spacing is when you give birth, then stay for 2 years without conceiving”* (FGD#6, women).

*“Child spacing gives time for a woman to rest from births”* (FGD#5, men).

*“Birth spacing is when you allow your child to grow before you give birth to another child”* (FGD#2 women).

*“Child spacing is when a woman is given some time to recover from her previous birth before she conceives again so that she stays healthy for her next birth. It also allows parents to plan for their children by providing them with proper feeding, medical care, and education” (FGD#9, men).*

For the participants, child spacing enabled the family to respond to primary needs and care for children (having enough material resources).

*“If you space children, you will have time to pay them [children] school but if you frequently produce, taking care of them becomes difficult (FGD# 7, women youth).*

*“When there is child spacing, children are allowed to grow without malnourishment" (FGD#1, men).*

It helped women to recover from birth and contribute to improving children’s development.

*“Child spacing allows children to grow very well such that they are healthy and bright (FGD# 9, men)*

*“There is a problem for those who do not space their children. Their children do not grow healthy and strong because they are not given enough time to breastfeed” (FGD# 10, women).*

Families who practice child spacing have a better productive life through achieving a career and better plan their family life. Table 2 described the results of the fuzzy cognitive mapping sessions. It highlights these three benefits of child spacing.

*“Women can have a career. “You can plan your life better (men youth, FGD# 4).*

*“Child spacing is living happily with your wife and few children (men youth, FGD# 8).”*

*“Child spacing is important. It helps you to take care of your children (men youth, FGD# 8).*

**Table 2:** The three most influential categories of child spacing

	Service providers	Men	Traditional midwives	Community health workers	Women	Male youths	Female youths
Sufficient material resources	1.00	1.00	0.56	1.00	1.00	1.00	0.39
Mothers and children healthy	0.33	0.40	1.00	0.28	0.06	0.43	0.17
Desire to have a better life	0.42	0.30	0.24	0.04	0.13	0.23	0.39

Women who use contraceptives without the husband's consent might face separation from their husbands. Divorce results in the loss of her home and access to land. After marriage, women in the Acholi culture live in the husband and in-laws' home. She does not own her own home and can access land only through her husband or sons. Land is the central asset for subsistence and a source of income. In the baseline survey, while 97% (224/231) of women reported earning money from agriculture work, 6% (13/214) stated that they are involved in land decisions.

“*If you go for family planning, it can lead to separation in the house (FGD#6, women).*

*“The man in the home is the one who can and should influence the use of land. The older men in a home are the ones to influence the use of land (FGD#9, women youth).*

*“It’s the man to decide about practicing child spacing because the woman came to the man’s home and should follow order from the man. “Lacoo aye won gang” meaning “The man is the owner of the home” (FGD#8, men youth).*

*“A woman cannot refuse your orders because you are the one who brought her to your home (FGD#5, men).*

Women who use contraceptive methods without the husband’s consent and refuse intimacy risk intra-partner violence. This can be a cause of short birth interval or amplified its effects:

*“When there is domestic violence, a woman will not use birth control methods like implants in fear of being beaten by her husband. That means she would not be able to space her children” (FGD#6, women).*

Both men and women acknowledged intra-partner violence, and forced intercourse is one violent manifestation. This results in a lack of child spacing:

*“The man threatens your life with a panga for you to sleep with him so you cannot space birth” (FGD# 7, women youth).*

*“Where there is domestic violence, the man drinks alcohol and refuses to take up child spacing” (FGD#2, women).*

*“A woman might choose to use implants or any other Western contraceptive, but when there is domestic violence, her husband would be rude and refuse for her to use such contraceptives so she would keep producing children continuously. Some men would also have intercourse with their wives forcefully even when the women are in their menstruation periods hence reducing child spacing” (FGD#9, women youth)*

*“Some men are slow to understand, so when their wives ask them for permission to use contraceptives, they do not accept. The women sneak and use them secretly, leading to domestic violence.” (DD#3, man).*

*“You can force your wife to sleep with you this way she conceives hence no child spacing” (FGD#8, men youth).*

*“I force my wife to sleep with me (FGD#8, men youth).*

In the survey, 36% (91/254) of women reported having suffered any form of mental/verbal abuse at home, and 19% (49/254) experienced physical violence in their last pregnancy (Table 3).

**Table 3:** Results from baseline survey on gender-based violence and family planning outcomes

Socio-demographic characteristics	Results from the survey % (n) or X̅ (SD, n)
Women interviewed	255
Age	29.2 (8.4, 255)
Number of children	3.3 (2.1, 248)
Woman’s education above primary	37.8 (249)
Woman did not attend school	4.4 (249)
Household head	
Husband	68 (255)
Parents/parents in law	21 (255)
Woman	7 (255)
Other	4 (255)
Husband is a farmer or manual worker	77 (255)
Woman or family own agricultural land	83 (255)
More than 2 acres of land	62 (204)
Woman is involved in decisions on land	6 (214)
Women is farmer or manual worker	74 (255)
Woman earns money from agricultural work	97 (231)
Husband is main financial provider at home	57 (255)
Woman contributes as one of the main financial sources at home	29 (255)
Children do not receive external financial support	95 (255)
Woman reported having enough food during the last week	62 (255)
Gender-based violence	
Woman experienced any form of mental/verbal abuse at home	36 (254)
Woman experienced physical violence	19 (254)
Child spacing and family planning	
Woman thinks it is worthwhile to use contraceptives to avoid/delay pregnancy	87 (254)
Contraceptives currently used	
Condom	0.4 (255)
Implant	18.4 (255)
Injection	22.7 (255)
IUD	1.2 (255)
Natural	1.6 (255)
Vaginal ring	2 (255)
None	39.6 (255)
Not applicable	14.1 (255)
Woman could use contraceptives if she wanted to do so	92 (255)
Factors can affect your decision on spacing children	
Does not trust in contraception	1 (255)
Fear of side effects	42 (255)
Male’s partner decision	10 (255)
Prefer traditional methods	2 (255)
No reason	116 (255)
Too young/being at school	10 (255)
Other	9 (255)
Most recent pregnancy outcomes	
The partner provided support during last pregnancy	70 (254)
Who paid for transportation to health facility	58 (234)
Husband	58 (234)
Women	30 (234)
Family	6 (234)
Volunteer/community member	6 (234)
Woman did not recognize any danger signs during pregnancy	38 (250)
Woman did not recognize any danger signs during labour	66 (249)
Woman did not have antenatal care	2 (256)
Average number of antenatal care visits	4.4 (1.4, 251)
Woman did not have blood test	29 (255)
Woman did not have urine test	54 (255)
Blurred vision and dizziness	68 (254)
Repeated headaches	70 (254)
Delivered in a health facility	90 (250)
Postpartum health outcomes	
Fever within the 6 weeks after delivery	42 (253)
Foul discharge from vagina within 6 weeks after delivery	21 (252)

In the following statements, the participants were discussing during a deliberative dialogue session how forced intercourse is a cause of lack of child spacing and what could be the consequences if a woman reports that her husband has raped her:

*“It is impossible for a woman to report to the authorities when she is raped by her husband because she fears she would be chased away by her husband’s relatives (DD# 1, woman).*

“*If a woman reports me for rape and I am arrested, she should just pack up her things and leave my home” (DD#1, man).*

While women stressed the challenge of discussing child spacing with their spouses and felt uncertain about their marriage, men highlighted their wives' lack of trust. This can result in intra-partner violence:

“*I tried to talk to my husband to accept for me to use family planning contraceptives, but he never accepted, so I started using the contraceptives secretly, (DD#2, woman).*

*“It is difficult to talk to the men of these days, but back in the days, we were able to convince our husbands easily about child spacing” (FGD#2, woman).*

*“When there is domestic violence, a man might suspect that his wife is having external affairs so he would decide to get her pregnant to stop such external affairs” (FGD#9, men).*

*“Family planning brings promiscuity. When a man leaves work, a woman sneaks out to sleep with another man because the effect of her going out won’t be seen. She cannot get pregnant by cheating on her husband” (FGD#2, men youth).*

*“When there is domestic violence, a woman would be cautious not to deliver many children with the man because she would not be sure about her continued stay with the man” (FGD# 10, women).*

*“When there is domestic violence, a woman can decide to sneak out and use implants to avoid giving birth to many children. This is because she would not be sure about her long stay with the man and would be afraid of raising children to single handily” (FGD#11, women youth).*

The context of poverty strengthened the tensions between spouses and can lead to intra-partner violence:

*“Domestic violence brings child spacing is when the man sells things in the house like foodstuffs without consent of the wife. She will think of going for child spacing” (FGD#5, men).*

From the survey, 87% (221/254) of women thought it worthwhile to use contraceptives to avoid/ delay pregnancy, yet 39.6% (101/255) did not use contraceptives. The most frequently used contraceptive was injections, 22.7% (58/255), followed by implants, 18.4% (47/255). Women described discomfort and concerns about using contraceptives. Some experienced side effects and complications after use. Some 42% (107/255) of women reported fear of side effects as a factor that can affect their decision to child space.

Women stressed their perceived impact of contraceptives on their children. Rumors circulating in their communities amplified feelings of anxiety and insecurity about contraception. Participants had limited information about the menstrual cycle (fertility period).

*“The women who use those contraceptives for long end up giving birth to very weak children when they decide to produce (FGD#1, women).*

*“I used the implants, but I suffered because I always felt something moving in my womb like a baby; I later found out that it was clotted blood which had to be removed at the hospital” (FGD#6, women).*

*“Those contraceptives are bad because I once used them, and I used to feel abdominal pains and constant vaginal bleeding until I stopped using them” (FGD#6, women).*

*“The modern contraceptives can make the users give birth to children with some missing body parts such as arms and legs (FGD1#1, men).*

*“Those who use implants sometimes experience a lot of weakness and dizziness. I have personally experienced that (FGD#5, women).*

*“People who use the implants can even end up dying because they sometimes suffer from complications” (FGD#1, men).*

Men shared the perceptions of the effects of contraceptives on women and children:

*“If you put Western medicine, you become barren” (FGD#8, men youth).*

*“Some people fear to use the Western contraceptives because we have seen women giving birth to children with swollen heads and missing arms or legs after using such contraceptive for long” (FGD#9, men youth).*

*The women complain so much that implants are bringing them illnesses (FGD#1, men).*

Both women and men described their knowledge about the menstrual cycle:

*“When there is domestic violence, a man would not respect his wife and have intimacy intercourse with her even when she is in her menstruation period which leads to pregnancies” (FGD#10, women).*

***“When a couple cooperate, a man would respect his wife when she tells him that she is in her menstruation period. That would increase child spacing” (FGD#4, men)***

Table 4 synthesizes the results of the FCM sessions on the causes of short birth intervals.

**Table 4:** The three most influential categories of short birth intervals

	Service providers	Men	Traditional midwives	Community health workers	Women	Male youths	Female youths
Gender dynamics	1.00	0.29	0.40	0.33	1.00	0.67	0.35
Fear of family planning side effects	0.65	1.00	1.00	1.00	0.59	0.73	1.00
Insufficient material resources	0.15	0.39	0.00	0.43	0.12	1.00	0.00

In the deliberative dialogue, communities proposed enhancing spousal communication, mutual understanding, increasing males' support in child spacing practices, tackling intra-partner violence, improving knowledge about contraceptive methods, and managing their side effects. The results from each parish deliberative dialogues converged. They recommended a home-based sensitization program undertaken by community health workers to discuss the causes underlying short birth intervals (lack of spousal communication, intra-partner violence, management of side-effects). The program will engage both women and men from households.

*“There should be good agreement between the men and the women (DD# 2, woman).*

*“The men should be involved in family planning” (DD#3, service provider).*

*“The men should start accompanying their wives when they go to seek the contraceptives. That would help both of them understand how the contraceptives would operate” (DD#2, man).*

*“Negotiations and peace talks should be arranged to reconcile the partners involved in domestic violence (DD#3, man).*

*“The decision to use family planning should be made by both partners” (DD#1, service provider).*

*“The best way to space children is by cooperation between a man and his wife (DD# 3, man).*

*“The women should be taught the importance of using contraceptives so that they become confident in using them (DD#2, woman).*

*“The women should regularly report to the health facility when using the contraceptives so that they are advised accordingly by the health workers on how to treat the side effects like severe vaginal bleeding (DD #2, service provider).*

## Discussion

Women, men, and youth in Nwoya district had a clear understanding of child spacing benefits. These benefits were difficult to translate into practice, however, because women were disempowered to make free choices on their fertility. Women either submitted to their husband’s decisions, or they had to use contraceptives covertly. Women who used contraceptives covertly risked paying a high price for doing so. They faced separation from their husbands and thus lost access to home and land. They were more likely to experience intra-partner violence. Women were anxious and felt insecure about using contraceptives because of the experience of side effects. They did not have enough information about how the menstrual cycle works.

Injectables and implants are predominant contraceptive methods in sub-Saharan Africa, accounting for 43% of contraceptives used. Changing in menstrual bleeding patterns (e.g., lighter or heavier bleeding, prolonged or irregular bleeding, or amenorrhoea) is extremely common for both contraceptive methods [25]. In line with our findings, several recent systematic and scoping reviews reported women’s concerns about side effects on the use of contraceptives [6, 7]. A 2018 scoping review found that 71 studies assessed women’s discontinuation, dissatisfaction, or non-use of contraception due to experience or perception of changing blood patterns [26]. In Northern Uganda, a cross-sectional study reported that fear of side effects (88.2%) and irregular periods (7.7%) are the most important reasons for not using family planning uptake services. The most common side effect mentioned was excessive bleeding, which led to discomfort and insecurity among women [27]. The 2018 scoping review included 100 studies, but only nine were from Africa, and only 13 had qualitative data [26]. There is a need for more in-depth work on the health and social implications of different contraceptive methods and reproducible co-design of interventions that consider women’s preferences and comfort.

Women in our study area needed land to produce food for their families. Ugandan women provide at least 75% of agriculture labor and legally own less than 10% of the land [28]. Land tenure and land ownership are passed through a patriarchal inheritance system. The last Ugandan DHS reported that only 38% of women owned a house alone or jointly compared with 54% of men, and 31% of women owned land alone or jointly compared with 48% of men [5]. In practice, women cultivate their husbands’ land, and after harvest, men take control of the resulting income. Using contraceptives covertly implies that women might lose access to land as their primary livelihood and income source.

Tensions around managing the financial resources generated by the harvest and the non-ownership of land by women amplify the spousal tensions, making marital relationships more fragile and, in turn, influencing birth interval. Many women reported they did not feel secure and were concerned about the sustainability of their marital relationships. A study in rural Uganda reported that having enough land to produce food for the family and feeling safe on family land were associated with contraceptives if the last pregnancy was intended [29].

The post-conflict situation of Northern Uganda adds a layer of complexity to the land issues. It increases inequalities in gender roles, male alcoholism consumption, and intra-partner violence [13, 30, 31]. In the last Ugandan DHS, 56% of women had experienced physical, sexual, or emotional violence by their current or most recent spouse/partner, and 46% of women said they were afraid of their current husband [5]. In our study, men and women related intra-partner violence to a lack of child spacing. Our study highlighted male disapproval and the lack of communication with spouses, in line with the evidence from other settings [6, 7]. Female youth were particularly fearful of their partners, and some discussed how their partners threatened them if they refuse intimate intercourse. Male youth openly declared to force their partners to have intimacy with them. Studies in Kenya, Niger, and the Philippines identified an association between intra-partner violence and covert family planning methods [31–33].

These findings demonstrate the value of engaging men to promote a culturally safe approach to child spacing, discussing harmful gender norms and practices. An intervention study that catalyzes men through community engagement reported increased use of contraceptive methods and a shift in gender norms such as spousal communication, controlling over cash earnings, and family planning self-efficacy [34].

### Strengths and limitations

Local team members who collected the data are Northern Ugandans from similar communities to those involved in the study. Non-Ugandan team members were not directly involved in collecting the data (FCM, FGD, and survey). One Canadian team member participated in deliberative dialogue sessions as a public health researcher, but she did not facilitate the sessions nor take any leadership role.

FCM and focus groups were gender- and age-stratified, and proceedings were not digitally recorded to encourage participants to be comfortable expressing their thoughts, ideas, and views on sensitive topics. The participatory approach creates safe spaces where participants can feel free to discuss and share their views. Participating communities were aware, however, that a Canadian organization funded the study. Although the funders were not at all linked to the local health services under discussion, it is conceivable this involvement introduced a social desirability bias. Local team members discussed the scope, objectives, and participatory nature of the project. Communities agreed to participate without financial incentives.

The fuzzy cognitive mapping and the focus group discussions findings helped build an emic grounded theory of short birth intervals. This theory informed emic solutions to promote culturally safe child spacing practices. During the deliberative dialogues, the research team validated the fuzzy cognitive mapping findings and the focus group discussions.

Our study disentangles the multiple and complex inter-related dynamics that lead to short birth intervals. It offers an understanding of how the economic context, gender power dynamics, inequitable gender land tenure, and ownership regulations, and the lack of considering women’s preferences and comfort on the use of contraceptive methods intersect and influence short birth intervals in a post-conflict setting.

The translation from Acholi to English may have lost or reduced the meanings of some concepts. Although the process did include older men and women, our failure to include designated elder groups prevents any discussion of generational differences. Our study described how rural women and men in Nwoya district think about short child spacing and the lack thereof. Therefore, generalized the findings to other settings are likely to be limited.

## Conclusions

The economic context, gender power dynamics, inequality, gendered land tenure and ownership, and the limited contraceptive supply limit women’s freedom to practice child spacing. The participants suggested a home-based sensitization program that discussed marital relationships (spousal communication, male support, intra-partner violence), contraception, and its side-effects management.

## Supplementary Information

**Additional file 1.** Thematic analysis for causes underlying short birth intervals.

**Additional file 2.** Thematic analysis for child spacing benefits.

**Additional file 3.** Pattern matching table for causes underlying short birth intervals.

**Additional file 4.** Pattern matching table for child spacing benefits.

## Data Availability

The datasets used and/or analysed during the current study are available from the corresponding author on reasonable request.

## Abbreviations

FCM: Fuzzy cognitive mapping
FGD: Focus group discussion
DD: Deliberative dialogues
