# Supplementary material for: Community views on short birth interval in Northern Uganda: a participatory grounded theory
Source: Reprod Health. 2021 Apr 28;18:88. doi: 10.1186/s12978-021-01144-5 (PMC8080315; doi:10.1186/s12978-021-01144-5)
Supplement: Supplementary file 3 — Additional file 3. Pattern matching table for causes underlying short birth intervals. [file 12978_2021_1144_MOESM3_ESM.docx]

Appendix2 a. Pattern matching table for causes underlying short birth intervals

| Causes/ stakeholder groups | Health workers | Men | Traditional midwives | Community health workers | Women | Young men | Young wome |
| --- | --- | --- | --- | --- | --- | --- | --- |
| Bad relationship between women and providers | 0.00 | 0.00 | 0.15 | 0.10 | 0.00 | 0.00 | 0.00 |
| Gender dynamics | 1.00 | 0.29 | 0.40 | 0.33 | 1.00 | 0.67 | 0.35 |
| Fear of family planning side-effects | 0.65 | 1.00 | 1.00 | 1.00 | 0.59 | 0.73 | 1.00 |
| Fear of losing culture | 0.00 | 0.14 | 0.00 | 0.00 | 0.00 | 0.00 | 0.00 |
| Frequent intercourse | 0.00 | 0.00 | 0.00 | 0.00 | 0.27 | 0.00 | 0.00 |
| Having the desired sex of the child | 0.00 | 0.00 | 0.00 | 0.05 | 0.00 | 0.00 | 0.00 |
| Ignorance about family planning | 0.35 | 0.18 | 0.25 | 0.14 | 0.00 | 0.80 | 0.00 |
| Insufficient material resources | 0.15 | 0.39 | 0.00 | 0.43 | 0.12 | 1.00 | 0.00 |
| Lack of male support | 0.00 | 0.00 | 0.35 | 0.00 | 0.53 | 0.00 | 0.17 |
| Lack of trust in governmental agencies | 0.00 | 0.00 | 0.00 | 0.00 | 0.00 | 0.00 | 0.13 |
| Mother has a disease | 0.00 | 0.00 | 0.00 | 0.00 | 0.18 | 0.00 | 0.00 |
| Society encourages to have many children | 0.65 | 0.36 | 0.45 | 0.67 | 0.00 | 0.53 | 0.04 |
