# Supplementary material for: Community views on short birth interval in Northern Uganda: a participatory grounded theory
Source: Reprod Health. 2021 Apr 28;18:88. doi: 10.1186/s12978-021-01144-5 (PMC8080315; doi:10.1186/s12978-021-01144-5)
Supplement: Supplementary file 4 — Additional file 4. Pattern matching table for child spacing benefits. [file 12978_2021_1144_MOESM4_ESM.docx]

Appendix 2b Pattern matching table for child spacing benefits

| Stakeholder groups/ causes | Service providers | Men | Traditional midwives | Community health workers | Women | Youth men | Youth women |
| --- | --- | --- | --- | --- | --- | --- | --- |
| Desire to have a better life | 0.42 | 0.30 | 0.24 | 0.04 | 0.13 | 0.23 | 0.39 |
| Mothers and children are healthy | 0.33 | 0.40 | 1.00 | 0.28 | 0.06 | 0.43 | 0.17 |
| Sufficient material resources | 1.00 | 1.00 | 0.56 | 1.00 | 1.00 | 1.00 | 0.39 |
| Preference for child gender | 0.00 | 0.00 | 0.00 | 0.00 | 0.00 | 0.00 | 0.09 |
| Fear of birth complications | 0.00 | 0.40 | 0.00 | 0.24 | 0.23 | 0.11 | 0.30 |
| Fear of family planning side effects | 0.00 | 0.00 | 0.00 | 0.16 | 0.23 | 0.00 | 0.00 |
| Frequent deliveries | 0.00 | 0.00 | 0.08 | 0.00 | 0.00 | 0.00 | 0.00 |
| Lack of Male support | 0.17 | 0.40 | 0.12 | 0.00 | 0.29 | 0.00 | 1.00 |
| Gender dynamics | 0.29 | 0.25 | 0.00 | 0.12 | 0.06 | 0.00 | 0.00 |
| Mother has a disease | 0.00 | 0.15 | 0.12 | 0.08 | 0.10 | 0.11 | 0.00 |
| Prestige to have many children | 0.00 | 0.00 | 0.00 | 0.00 | 0.03 | 0.00 | 0.00 |
| Use of family planning methods | 0.42 | 0.00 | 0.04 | 0.24 | 0.00 | 0.00 | 0.13 |
